# High performance and low temperature coal mine gas sensor activated by UV-irradiation

**DOI:** 10.1038/s41598-018-34707-x

**Published:** 2018-11-02

**Authors:** Salimeh kimiagar, Vahid Najafi, Bartlomiej Witkowski, Rafal Pietruszka, Marek Godlewski

**Affiliations:** 1 Nano Research Lab (NRL), Department of Physic, Islamic Azad University, Central Tehran Branch (IAUCTB), Tehran, Iran; 2 Young Researchers and Elite Club, Central Tehran Branch, Islamic Azad University, Tehran, Iran; 3 Institute of Physics, Polish Academy of Sciences Al. Lotnikow 32/46 PL-02668, Warsaw, Poland

## Abstract

In this work, well-aligned vertical ZnO nanorod (ZnO NRs) on p-type Si substrate was fabricated by a microwave-assisted hydrothermal reactor to study the coal mine methane (CMM) gas sensing properties. The XRD diffraction peaks and Raman spectra of the ZnO NRs confirmed the hexagonal wurtzite structure with strong preferential orientation along the c axis and well crystal quality. SEM analysis showed NRs with 100 nm average diameter and ~600 nm length. The variations of the sensor electrical resistance in the presence of CMM were investigated at different gas concentrations and various temperatures in the dark and under UV light. The selectivity and response time of the sensor to CMM gas were improved under UV irradiation. The optimal operating temperatures were 225 °C and 100 °C in dark and exposing UV-irradiation, respectively. Also the response of ZnO NRs sensor under UV excitation in humid condition was higher. The sensor was more selective to CMM than CO_2_. The sensor stability was considered by repeating CMM detection for 90 days.

## Introduction

Coal mine methane (CMM) which released generally during and after mining operations is the main reason for the explosion in coal mines^1,2^. Coal mining is considered much more hazardous than hard rock mining. Incident reports show that the presence of minor amount (about 1–3%) of CMM in the underground mines atmospheres causes increasing the explosion risk to about 75%^3,4^. Hence, timely gas detection in underground mines and rapid ventilation is essential for coal miner safety^5^. Semiconductor metal oxide gas sensors thanks to their unique advantages such as fast response, good recovery speed, low cost, and high reproducibility, can be appropriate option to detect the toxic and explosive gases^6,7^. Among the various semiconductor metal oxide, one-dimensional Zinc oxide (ZnO) nanostructures especially Zinc oxide nanorods (ZnO NRs) due to the large surface to volume ratio, persistent photoconductivity, intrinsic oxygen defects and other excellent electrical and optical properties are one of the most suitable material for gas sensing^8–10^. However, in spite of all the advantages which considered for ZnO NRs as a gas sensor, they also have a high operating temperature which is significant demerit^11^. This is important since applying high temperature to the sensor in the presence of explosive gases may cause an explosion. Therefore, have been great efforts to improve the operating temperature of sensors based on ZnO NRs such as doping with noble metals^12^, surface decoration^13^, encapsulation process^14^, applying the electrostatic field^15^ and UV-irradiation technique^16,17^. Among these techniques, the most promising expected method is using UV-irradiation technique for achieving to practical low-temperature ZnO NRs gas sensors. Over the previous decade, many ZnO NRs arrays syntheses techniques have been reported^18–22^. However, it is required to fabricate high-quality and high-density vertical ZnO NRs by using different techniques.

In this work, we have synthesized well-aligned high-density vertical ZnO nanorods on p-type Si (100) substrates by both ALD technique and hydrothermal method. The prepared ZnO nanorod sensor was tested for coal mine gas detection. The advantage of our approach is achieving fast nanorods growth at low temperature and at atmospheric pressure.

## Experimental Details

### Samples preparation

The p-type Si substrate with dimensions of 3 × 3 × 0.05 cm^3^ (Si (100) wafer, Sigma Aldrich, contains boron as a dopant) was cleaned supersonically by toluene, acetone, and ethanol, then oxidized in a horizontal tube furnace with a flow of oxygen gas at 1200 °C. ZnO as a seed layer was deposited on Si/SiO2 substrates in the ALD reactors (Cambridge NanoTech) by repeating 13 ALD cycle at 100 °C. In the process, Diethylzinc (DEZ, CAS Number 557-20-0) and deionized water were used as a Zinc and oxygen precursors, respectively. After ALD nanoseeds growth, the samples were transferred to a microwave-assisted hydrothermal reactor (ERTEC Wafer) together with a solution consisting of Zinc acetate dissolved in deionized water(pH = 7.5). The substrate and the solution were heated to 50 °C and maintained at this temperature for 1 min. In order to clean the NRs surface from products of the reaction, the substrate with ZnO NRs was rinsed with isopropanol and annealed in the ALD reactor at a temperature of 200 °C for 2 hours. More details about ZnO NRs growth can be found in ref.^19^.

### Sensing setup

**Figure 1:**
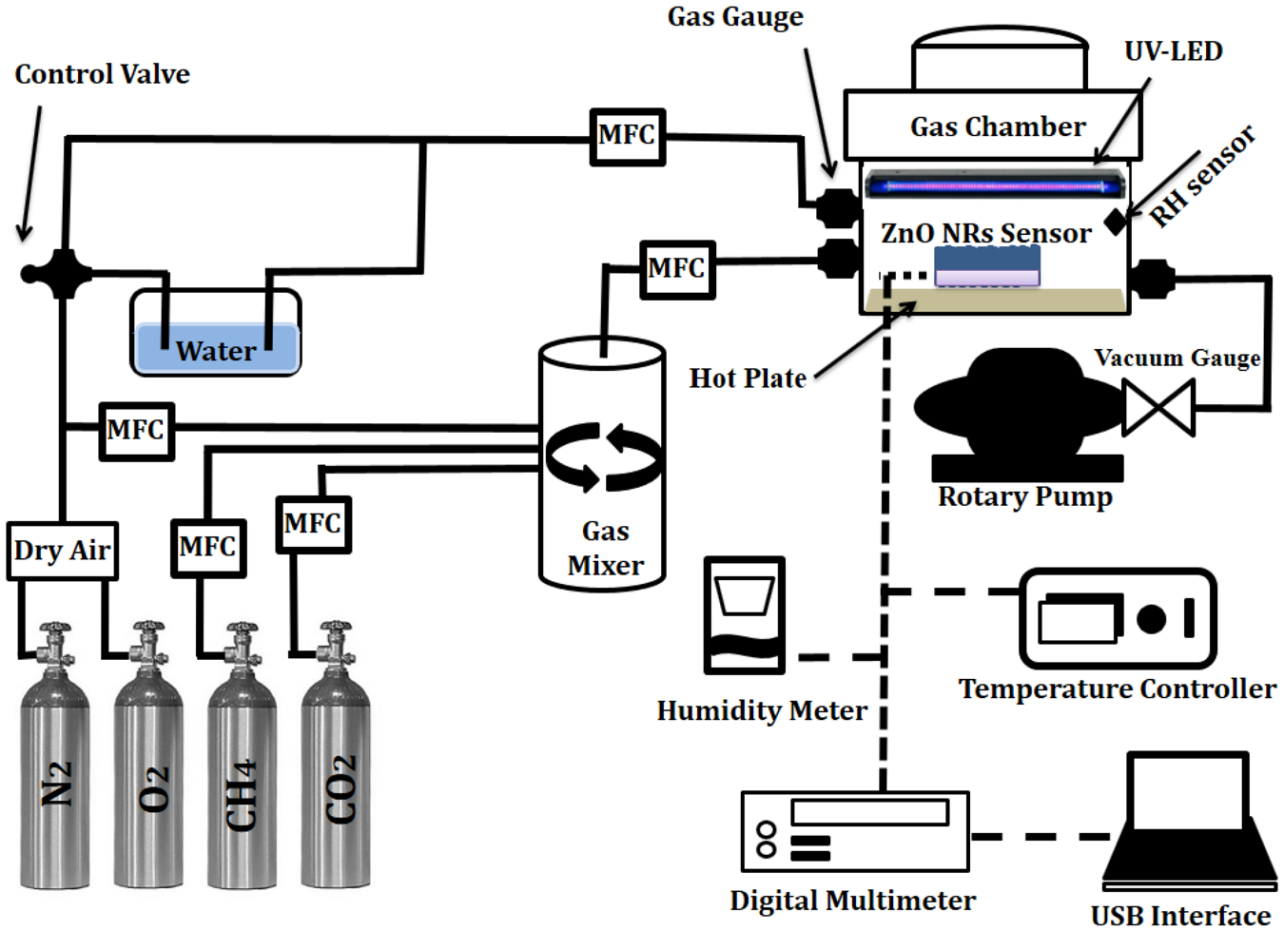
Schematic diagram of gas sensing setup.

The sensor has been fabricated by e-beam deposition of a pair of interdigital gold thin film electrodes on the samples. To investigate sensing properties, the sensor was placed in a chamber which provided temperature up to 400 °C for the sample. A series of UV-LEDs (λ = 365 nm) upon sample was used for ultraviolet light illumination. The power of UV illumination determined by the distance between the UV-LEDs and gas sensor. The electrical resistance of the sample was measured by an accurate digital multimeter (Hioki- IM3536). The target gas concentration in the chamber was determined using the dry air mixture which controlled by five mass flow controllers (MFC, AliCat Scientific). The variations of the sensor electrical resistance were investigated in the air and in the presence of CMM at different gas concentrations and various temperatures in the dark and under UV light. According to coal mine methane gas quality analyses, the CMM was considered a mixture of methane (CH_4_) and carbon dioxide (CO_2_) gases with ratio 19:1 respectively. Figure 1 shows the CMM gas sensor experimental setup.

## Results and Discussion

### Morphological and Nano structure analyses

**Figure 2:**
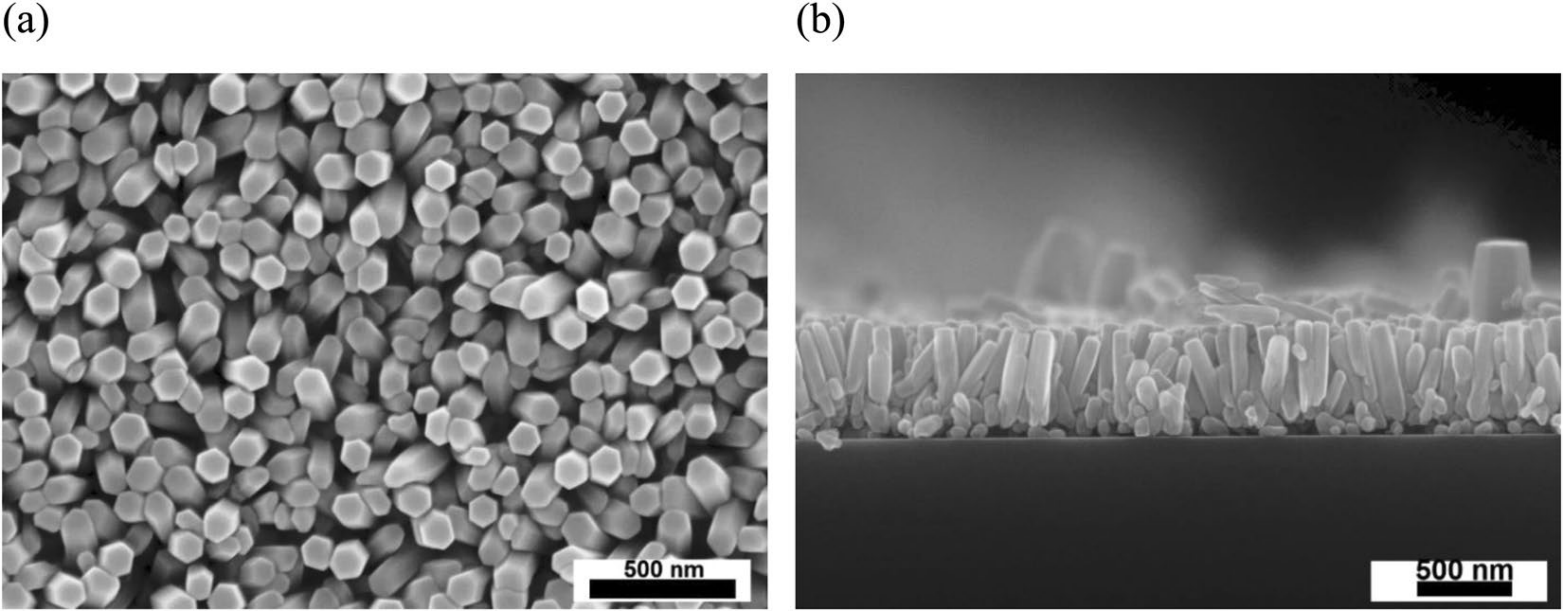
SEM images of (**a**) ZnO NRs, (**b**) ZnO NRs cross-section.

The surface morphology of the samples was investigated by a scanning electron microscope (SEM, CamScan-MV2300). Figure 2a reveals high- density ZnO NRs have wurtzite structure with the average diameter of 100 nm. According to Fig. 2a the calculated number density of ZnO NRs is ~6 × 109 cm^−2^ that significantly higher than previous literature^23,24^. The c-axis preferred crystal orientation tends to have the well-aligned and high-density ZnO NRs. The well-aligned vertical NRs with ~600 nm length can be seen clearly in cross-section of the sample (Fig. 2b).

**Figure 3:**
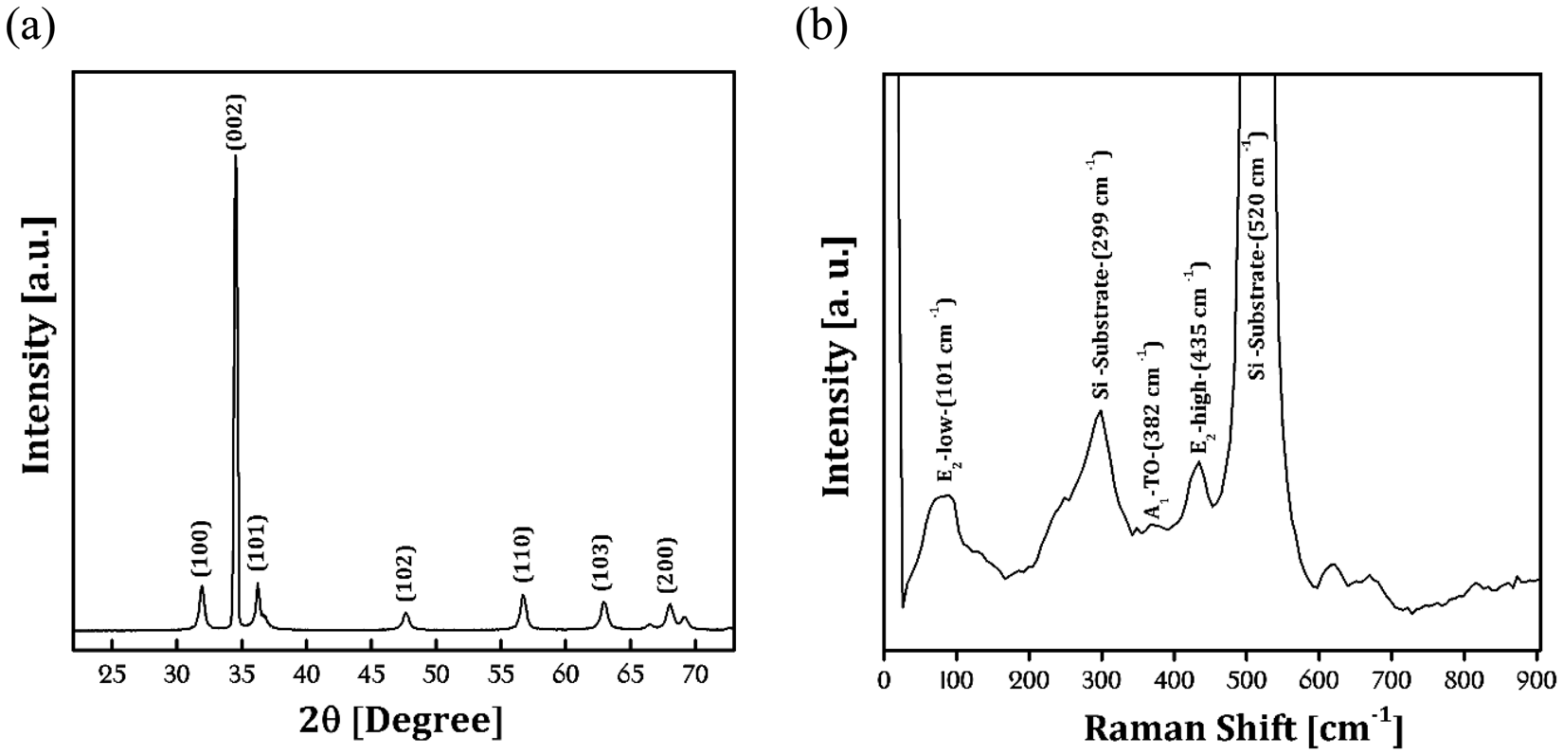
X-ray diffraction pattern (**a**) and Raman spectra (**b**) of ZnO NRs sample.

Figure 3a displays the X-ray diffraction pattern (XRD, Philips-MPD) of the sample. The main diffraction peaks corresponding to (100), (002) and (101) planes of ZnO NRs are attributed to hexagonal wurtzite structure (JCPDS Card No. 80-0074). The (002) diffraction peak of the ZnO NRs confirms the hexagonal wurtzite structure with strong preferential orientation along the c axis. In the other words, the results indicate that the ZnO nanorod arrays are highly aligned on Si (100) substrate with c-axial growth direction as confirmed by SEM analysis. Figure 3b exhibits the Raman spectra of ZnO NRs sample on Si substrate. According to the main optical modes in ZnO NRs, different scattering intensities at 101 cm^−1^, 383 cm^−1^ and 435 cm^−1^ are attributed to the E_2_ (low), A_1_ (TO) and E_2_ (High) modes, respectively. The E_2_ (low) mode is associated with the vibration of the heavy Zn sublattice and the E_2_ (high) mode involves only the oxygen atoms^25,26^. The presence of sharp mode at 435 cm^−1^ corresponds to the E_2_ mode conforms wurtzite hexagonal phase of ZnO with very well crystal quality. The lattice defects, Zinc interstitial and oxygen vacancy typically determined by transverse-optical (TO) and longitudinal-optical (LO) mods^27^. Therefore, some soft TO and LO modes which occurred at 333 cm^−1^, 383 cm^−1^, and 661 cm^−1^ may be related to the oxygen vacancy. The peaks at 299 cm^−1^ and 520 cm^−1^ are associated with Si/SiO2 substrate.

**Figure 4:**
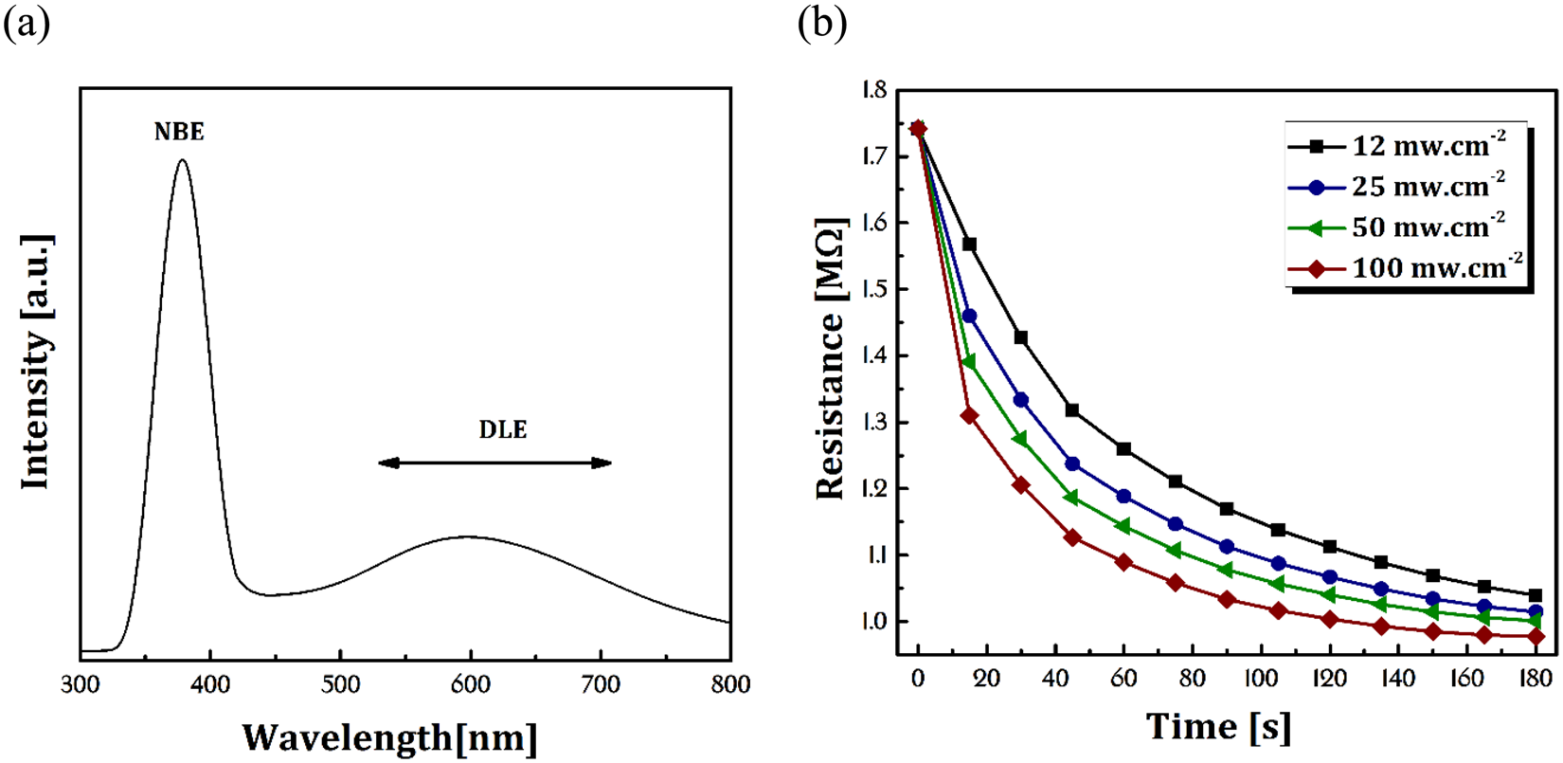
(**a**) PL spectrometry of ZnO NRs sample (**b**) resistance vs time plot of ZnO NRs in various UV light power.

The optical properties of the ZnO NRs were investigated by photoluminescence spectroscopy (PL, PerkinElmer). Figure 4a shows the PL spectra of ZnO NRs arrays measured at room temperature. The spectra contain two peaks, one in the UV region at ~378 nm affiliated to the near-band-edge (NBE) and one at ~595 nm related to the deep level emission (DLE) of ZnO NRs^28^. It is known the high-intensity NBE peak implies to the high-crystal quality of ZnO NRs and excellent optical properties in UV region. The appropriate NBE peak intensity in our sample shows it is a good option to use for UV-activated gas sensors. Furthermore, the broad DLE peak in the visible region appeared due to common lattice defects associated with oxygen and zinc vacancies. From the literatures^29–31^, these defects and particularly oxygen vacancy located at the surface of ZnO NRs, have an important role in gas sensor operation. The sample resistivity variations vs time under UV-irradiation with various powers are shown in Fig. 4b. Excitation by UV creates pairs of electron-hole in ZnO NRs which interacts with the adsorbed oxygen on the surface. This interaction during several stages leading to the reduction in electrical resistance of NRs. So, increasing the UV light power which means increasing the number of photons in UV region, can accelerate this resistance reduction. It should be noted that the mechanism of conductivity of ZnO NRs when exposed to CMM gas in dark and under UV-irradiation is explained in next section by details.

#### B- Sensing Analyses

To obtain the response of the ZnO NRs sensor for CMM detecting, the electrical resistance was measured in dry air and in the presence of 250–2000 ppm concentrations of CMM in the temperature range of 25–250 °C under UV excitation with the power of 100 mW.cm^−2^ and in dark. The response of n-type semiconductor for reducing gas (CH_4_) and oxidizing gas (CO_2_) is defined respectively as:

1 $$\begin{array}{l}{\rm{Response}}\,( \% )=\frac{|{R}_{a}-{R}_{g}|}{{R}_{a}}\times 100,{\rm{For}}\,{\rm{reducing}}\,{\rm{gas}}\\ {\rm{Response}}\,( \% )=\frac{|{R}_{g}-{R}_{a}|}{{R}_{g}}\times 100,{\rm{For}}\,{\rm{oxidizing}}\,{\rm{gas}}\end{array}$$

where *R*_*a*_ is the sample resistance measured at ambient environment while *R*_*g*_ is that under the test gas. The mechanism of electrical resistance variations of ZnO NRs exposed to CMM and air in dark and UV light can describe as follow. As previously mentioned ZnO NRs have defect states such as zinc interstitials and oxygen vacancies on its surface. The air oxygen molecules can be adsorbed on the vacancies at NRs surface due to chemical potential energy difference. After adsorption, the O_2_ molecules capture one or two electrons from the ZnO conduction band to reach the ${{\rm{O}}}_{2}^{-}\,{\rm{or}}\,{{\rm{O}}}_{2}^{2-}$ form and stay stable (g denotes to gas)^32,33^.

2 $$\begin{array}{c}{{\rm{O}}}_{2}({\rm{g}})+{{\rm{e}}}^{-}\to {{\rm{O}}}_{2}^{-}({\rm{ad}})\\ {{\rm{O}}}_{2}({\rm{g}})+2{{\rm{e}}}^{-}\to {{\rm{O}}}_{2}^{2-}({\rm{ad}})\end{array}$$

Therefore, the surface of ZnO NRs in common state has the chemisorbed oxygen ions $({{\rm{O}}}_{2}^{-}({\rm{ad}})\,{\rm{or}}\,{{\rm{O}}}_{2}^{2-}({\rm{ad}}))$ which are thermally stable and is difficult to remove from the surface. When ZnO NRs sensor is exposed to CMM in dark, these oxygen ions, react with the CMM and released 4 or 8 electrons to the conduction band (Eq. 3). This reaction leads to a smaller *R*_*g*_ than *R*_*a*_ and resulting higher response^34^.

3 $$\begin{array}{c}{{\rm{CH}}}_{4}({\rm{g}})+2{{\rm{O}}}_{2}^{-}({\rm{ad}})\to {{\rm{CO}}}_{2}({\rm{g}})+2{{\rm{H}}}_{2}{\rm{O}}({\rm{g}})+4{{\rm{e}}}^{-}\\ {{\rm{CH}}}_{4}({\rm{g}})+2{{\rm{O}}}_{2}^{2-}({\rm{ad}})\to {{\rm{CO}}}_{2}({\rm{g}})+2{{\rm{H}}}_{2}{\rm{O}}({\rm{g}})+8{{\rm{e}}}^{-}\end{array}$$

But as mentioned, chemisorbed oxygen ions are in thermally stability and strongly attached due to large adsorption energy. So, the above reaction requires high temperatures which called operating temperature. Hence using the UV-irradiation technique could be very effective. The UV photons create a pair of electron-hole in ZnO ($hv\to {h}^{+}(hv)+{e}^{-}(hv)$). In the ambient air, when ZnO NRs are illuminated by UV light the photogenerated hole interacts with the adsorbed oxygen ions and this oxygen can be desorbed by the NRs surface according to the following reaction^35^.

4 $$\begin{array}{c}{h}^{+}(hv)+{{\rm{O}}}_{2}^{-}({\rm{ad}})\to {{\rm{O}}}_{2}({\rm{g}})\\ 2{h}^{+}(hv)+{{\rm{O}}}_{2}^{2-}({\rm{ad}})\to {{\rm{O}}}_{2}({\rm{g}})\end{array}$$

At the same time, the photogenerated electron joins to the conduction band and leads to a reduction in electrical resistance of NRs (See Fig. 4b). By reducing the number of oxygen ions on the surface due to Eq. 4, the balance of energy causes the photogenerated electron tend to surface and react with the ambient oxygen molecules^35^.

5 $$\begin{array}{c}{{\rm{O}}}_{2}({\rm{g}})+{{\rm{e}}}^{-}(hv)\to {{\rm{O}}}_{2}^{-}(hv)\\ {{\rm{O}}}_{2}({\rm{g}})+2{{\rm{e}}}^{-}(hv)\to {{\rm{O}}}_{2}^{2-}(hv)\end{array}$$

**Figure 5:**
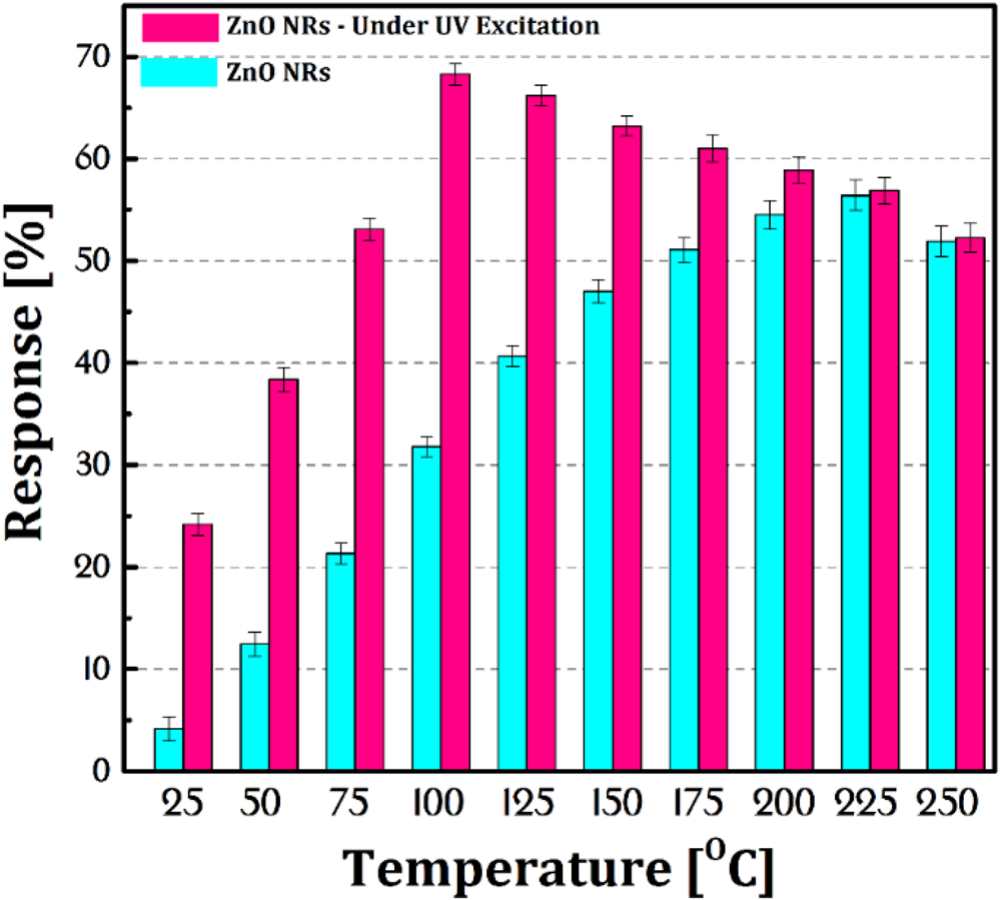
The response bar diagram of the ZnO NRs sensor to CMM gas under UV light and without excitation at different operating temperatures.

Then the reduction in electrical resistance of NRs is stopped and electrical resistance stays in a stable state due to equality in oxygen adsorption and desorption rate. The photo-induced oxygen ions ${{\rm{O}}}_{2}^{-}$(hv) are weakly bound to ZnO but the chemisorbed oxygen ions are strongly attached to the ZnO surface. The difference between sensor state after reaction 2 and 5 is that these oxygen ions $({{\rm{O}}}_{2}^{-}(hv)\,{\rm{or}}\,{{\rm{O}}}_{2}^{2-}(hv))$ are feeble bounded and can easily react with the CMM in lower temperature according to Eq. 3 ^35^. Figure 5 shows the sensor’s response to CMM (1000 ppm) at different operating temperatures. The optimal operating temperature was 100 °C and 225 °C for ZnO NRs under 100 mW.cm^−2^ UV-irradiation and in dark, respectively. Although as seen, the sensor at room temperature (25 °C) under UV excitation have an acceptable response (~23%) which makes it possible to use it in coal mines. It is clear using the UV-irradiation technique leads to improve the response of sensor at a lower temperature. By temperature increasing up to 100 °C, in addition to the photogenerated oxygen ions, the chemisorbed oxygen ions will attend in reaction also which will increase the response. After this temperature, the recombination rate increases due to electron-phonon coupling and creation rate of photogenerated oxygen ions decreases. Finally, at the temperature of 225 °C, the response of ZnO NRs sensors under UV excitation and dark are the same.

Alongside CMM in coal mines there are other gases such as CO_2_ which may disrupt the CMM gas detection process. Therefore the selectivity to CMM gas is an important parameter for sensor using in coal mines. The selectivity of the sensor is defined as the ratio of its response to a CMM to that of CO_2_ as:

6 $${\rm{Selectivity}}=\frac{{\rm{Response}}\,{\rm{to}}\,{\rm{CMM}}}{{\rm{Response}}\,{\rm{to}}\,{{\rm{CO}}}_{2}}$$

**Figure 6:**
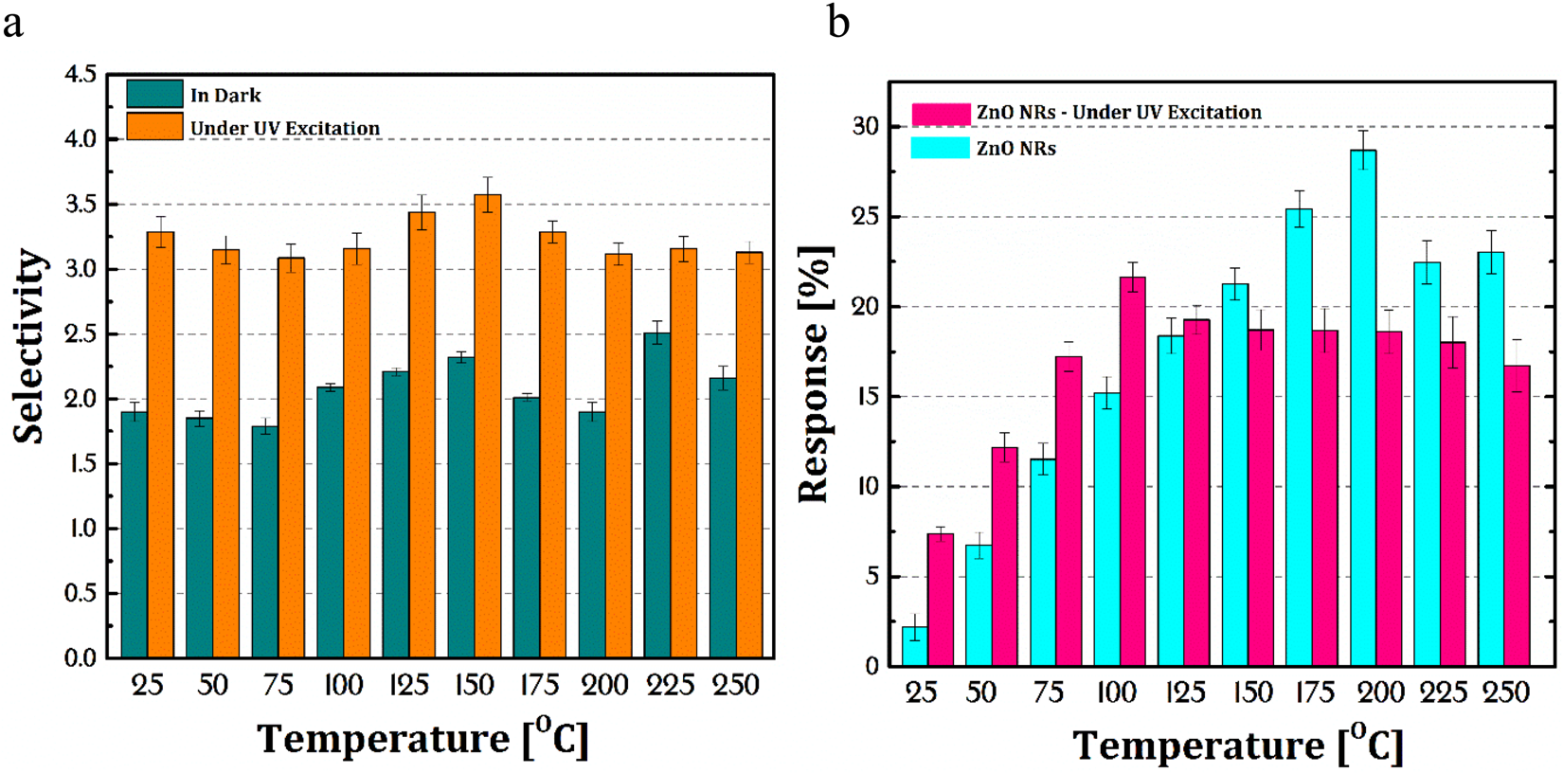
(**a**) The selectivity of CMM compared to CO_2_ gas in dark and under UV light at different temperatures and (**b**) the response bar diagram of the ZnO NRs sensor to CO_2_ gas.

Figure 6a shows the bar diagram of the selectivity of the ZnO NRs sensor to CMM compared to CO_2_ gas under dark and UV excitation at the concentration of 1000 ppm for both gases. As it is clear, under UV excitation the selectivity at all temperatures enhanced almost double.

The response bar diagram of the ZnO NRs sensor to CO_2_ gas in dark and under UV light at different temperatures is shown in Fig. 6b. As it is obvious, the response of the sensor to CO_2_ gas is lower than CMM gas in dark and UV at all temperatures. In addition, the sensor response under UV is less than the response in the dark. Briefly, this behavior can be can be justified by describing the reaction of CO_2_ with adsorbed oxygen ions on the ZnO NRs surface. First, the CO_2_ catchs an electron from the conduction band and is converted to ion and then is reacted with adsorbed oxygen ions as follows^36^:

7 $$\begin{array}{c}{{\rm{CO}}}_{2}({\rm{g}})+{{\rm{e}}}^{-}\to {{\rm{CO}}}_{2}^{-}\\ 2{{\rm{CO}}}_{2}^{-}+\,{{\rm{O}}}_{2}^{-}({\rm{ad}})+{{\rm{e}}}^{-}\to 2{\rm{CO}}({\rm{g}})+2{{\rm{O}}}_{2}^{2-}({\rm{ad}})\end{array}$$

Depend on the Eqs 7 and 3, the number of electrons released upon exposure to CMM is more than electrons which CO_2_ catchs in the conduction band, hence sensor presents higher response to CMM gas. In other hand, the final products at the end of the Eq. 7 are the CO gas and oxygen ions which may be reacting again as^37^:

8 $${\rm{CO}}({\rm{g}})+{{\rm{O}}}_{2}^{2-}({\rm{ad}})\to {{\rm{CO}}}_{2}({\rm{g}})+\frac{1}{2}{{\rm{O}}}_{2}({\rm{g}})+2{{\rm{e}}}^{-}\,\,$$

Thus, the electron comes back to the conduction band and response is decreased. When the sensor is excited by UV irradiation, operating temperature comes down owing to lower adsorption energy of ${{\rm{O}}}_{2}^{2-}(hv)$ compare to ${{\rm{O}}}_{2}^{2-}({\rm{ad}})$ in Eq. 7, as previously mentioned. In addition, under UV exciton the CO_2_ molecules photolysis to CO^38,39^ and reduces response of the sensor due to higher probability of the Eq. 8.

**Figure 7:**
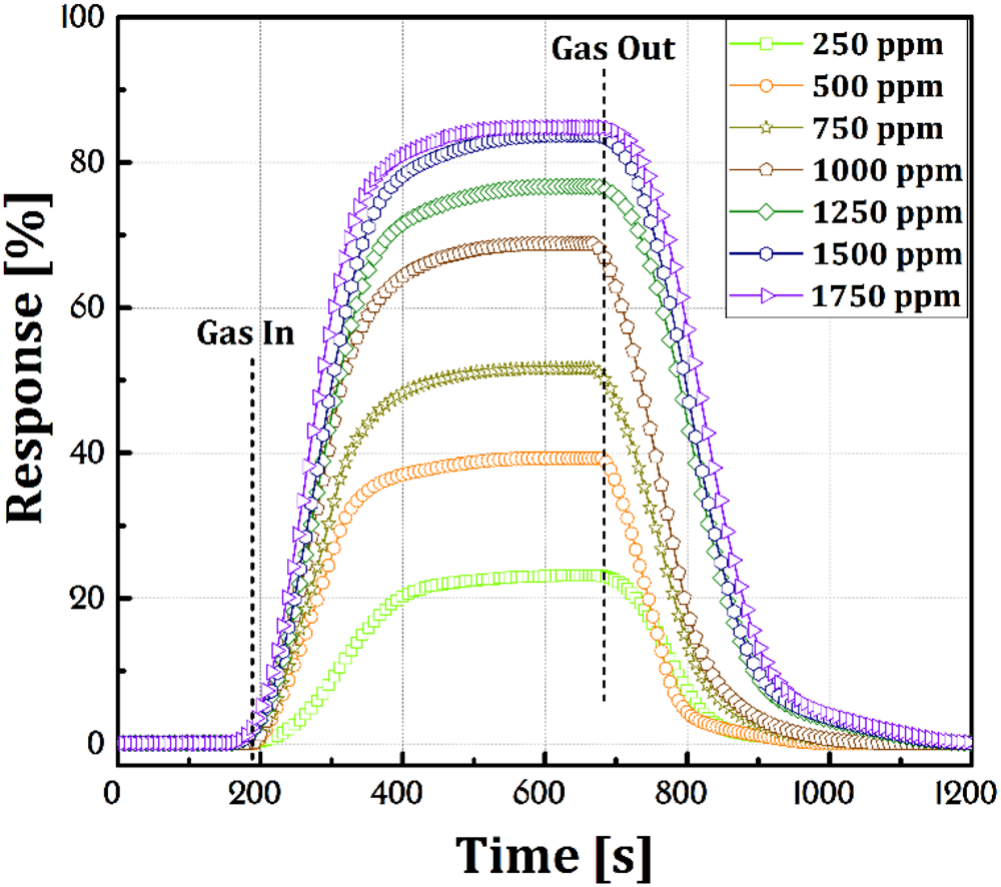
Dynamic variation of the response of ZnO NRs sensor under UV excitation that exposed to CMM gas with different concentrations at 100 °C operating temperature.

Figure 7 shows the dynamic variation of the response of ZnO NRs sensor exposed to CMM gas with different concentrations (250–1750 ppm) under UV-irradiation (100 mW.cm^−2^) at 100 °C operating temperature. According to Fig. 7, after exiting CMM from the test chamber, the resistance value is fully recovered to initial electrical baseline, which is demonstrating excellent stability, recovery, and reproducibility of the sensor. The sensing data are listed in Table 1.

**Table 1:** The recovery and response time for ZnO NRs sensor under UV excitation that exposed to CMM gas with different concentrations at 100 °C operating temperature.

Concentration (ppm)	250	500	750	1000	1250	1500	1750
Response Time (s)	210	150	102	82	68	62	57
Recovery Time (s)	142	120	158	163	182	224	241

**Figure 8:**
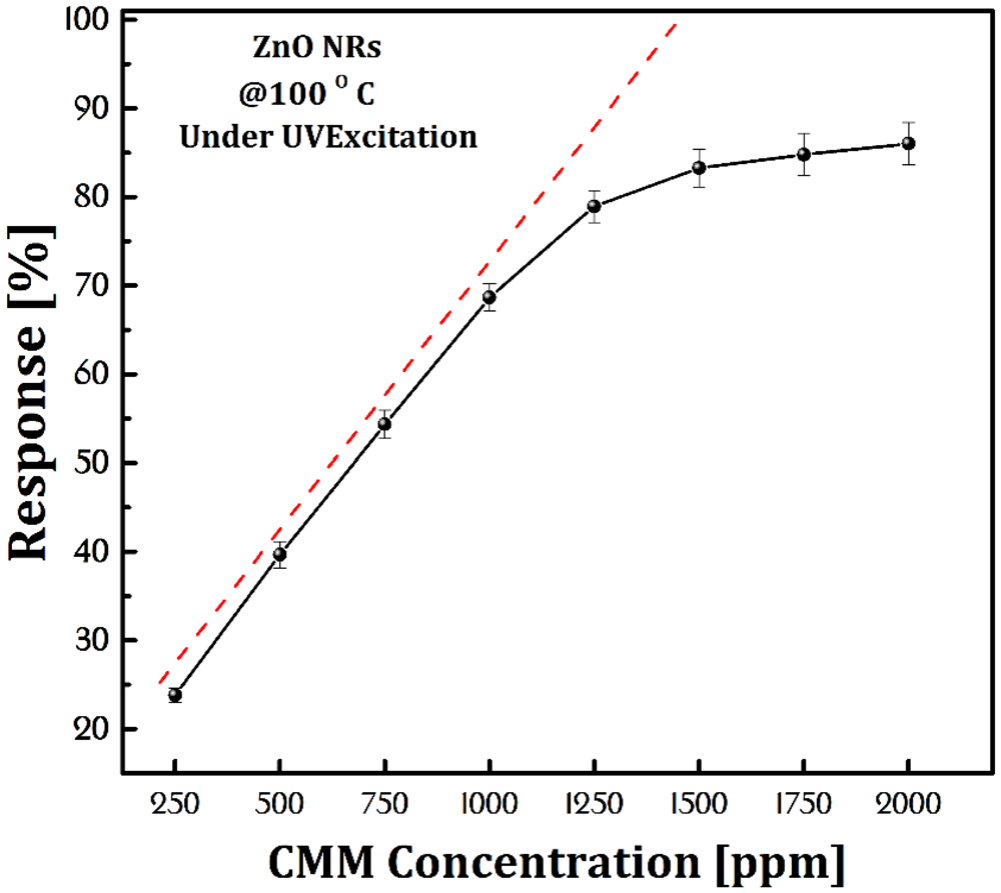
Limit detection of CMM gas with different concentrations by ZnO NRs sensor under UV excitation at 100 °C operating temperature.

The response time of a sensor is defined as a period of time which sensor surface atoms reacted to the target gas atoms and increase the concentration of carriers. In return, the recovery time refers to the period of time when the sensor returns to its original state in the absence of the target gas. The response and recovery time of the sensor exposed to 250–1750 ppm CMM under 100 mW.cm^−2^ UV-irradiation at 100 °C operating temperature were 210–57 s and 142–241 s respectively (Table 1). The sensor was saturated at 1500 ppm concentrations (Fig. 8). An increase in the gas concentration provides more gas atoms to be adsorbed on the sample surface which in turn causes an increase in the recovery time and a decrease in the response time. When all of the surface atoms are reacted with the target gas atoms the sensor is saturated. Nunes *et al*. reported a ZnO-based methane sensor at operating temperature of 373 °C but without any information on the dynamic response characteristics^40^. Bhattacharyya *et al*.^41^ results showed the ZnO sensor response time 65–106 s at operating temperature 150 °C for methane. Zhou *et al*.^42^ reported 41 s response time at 275 °C working temperature for the methane sensing of ZnO. Zhang *et al*.^9^ presented 25 s response time at 350 °C employed the temperature of the ZnO sensor. Chen *et al*.^43^ studied the methane sensing by ZnO nanostructure. Their ZnO sensing investigation showed 810 s response time at 300 °C. The results of this study in Table 1 show optimum value for response and recovery time compared to previous reports.

**Figure 9:**
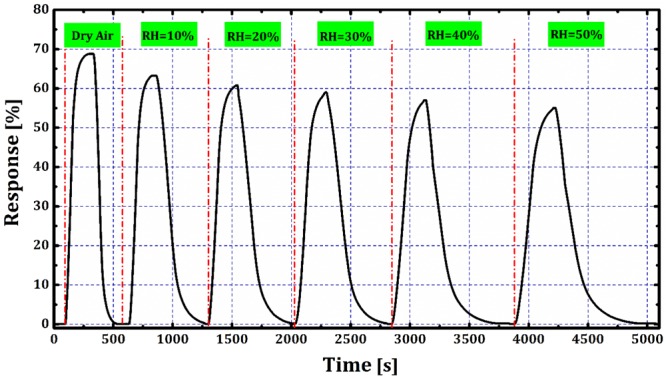
RH effect on CMM gas detection with different relative humidity by ZnO NRs sensor under UV excitation at 1000 ppm CMM and 100 °C operating temperature.

One of the most challenges to metal oxide gas sensors development is poor selectivity to gases in relative humidity (RH, defined as the percentage of water vapor in the air compared to the saturation level)^44^. High resistance to RH is an essential for sensors which used in coal mines. Humidifying in our study achieved by a mixture of dry air with humid air which controlled by the manual regulator (See Fig. 1). The error of humidity value was determined ±0.3% RH. Figure 9 shows CMM detection by the sensor at 1000 ppm and 100 °C operating temperature under UV excitation in 0–50% of RH. As can be seen, the response of ZnO NRs sensor to CMM gas has no significant change in humid (%50) condition under UV excitation which is extraordinary. The reason for this behavior can be described as follows.

When the sensor is in the dark at operating temperature, the interaction of water in the interface of water vapor and ZnO NRs can be written^45,46^:

9 $$\begin{array}{c}{{\rm{H}}}_{2}{\rm{O}}({\rm{g}})+{{\rm{O}}}_{2}^{-}({\rm{ad}})+2{\rm{Zn}}\to 2({\rm{Zn}}-{\rm{OH}})+{{\rm{e}}}^{-}\\ {{\rm{H}}}_{2}{\rm{O}}({\rm{g}})+{{\rm{O}}}_{2}^{2-}({\rm{ad}})+2{\rm{Zn}}\to 2({\rm{Zn}}-{\rm{OH}})+2{{\rm{e}}}^{-}\end{array}$$

Since the number of water molecules is much higher than CH_4_ molecules, it is obvious that the probability of reactions in Eq. 3 compared with Eq. 9 decreases and the response is reduced. So the main barrier of metal oxide-based sensors is the dependence of their response and resistance to the humidity^47^. When the sensor is excited by UV irradiation, the required energy for the following reaction on the sensor surface is provided by the UV photons^48^:

10 $${{\rm{H}}}_{2}{\rm{O}}({\rm{v}})+{{\rm{e}}}^{-}+{{\rm{h}}}^{+}\to {{\rm{H}}}_{2}({\rm{g}})+{{\rm{O}}}_{2}({\rm{g}})$$

This creates H_2_ as a reducing gas. As mentioned earlier, when ZnO NRs sensor is exposed to reducing gas, due to the reaction with oxygen ions several electrons are released to the conduction band as follows^49^:

11 $$\begin{array}{c}{{\rm{H}}}_{2}({\rm{g}})+{{\rm{O}}}_{2}^{-}(hv)\to 2{{\rm{H}}}_{2}{\rm{O}}+{{\rm{e}}}^{-}\\ {{\rm{H}}}_{2}({\rm{g}})+{{\rm{O}}}_{2}^{2-}(hv)\to 2{{\rm{H}}}_{2}{\rm{O}}+2{{\rm{e}}}^{-}\end{array}$$

According to the previous reports^50–52^, the H_2_ and CO are much higher reducing gases compare to CH_4_ and quickly are reacted to oxygen ions which cause not to decrease the response. Therefore the response of ZnO NRs sensor under UV excitation in humid condition has higher stability.

**Figure 10:**
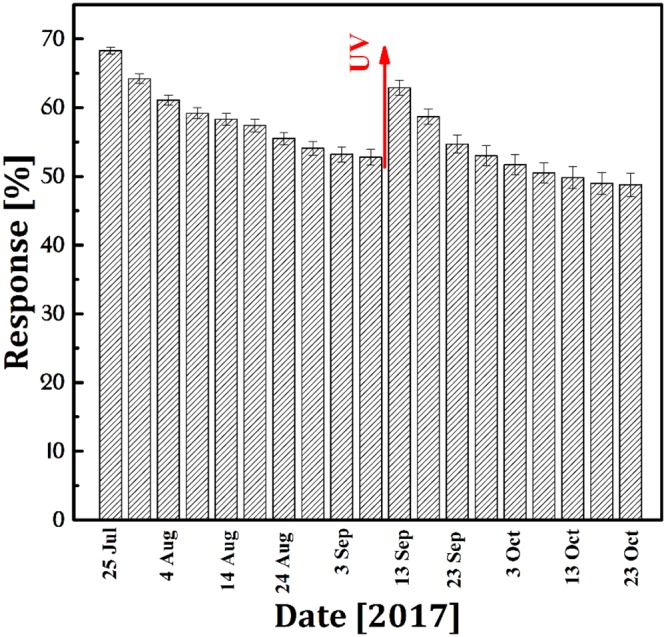
The ZnO NRs sensor stability under UV excitation at 1000 ppm and 100 °C operating temperature every 5 days.

Finally, the sensor stability was investigated by repeating CMM detection by the sensor at 1000 ppm and 100 °C operating temperature under 100 mW.cm^−2^ UV excitation in every 5 days for 90 days. Figure 10 shows the response of ZnO NRs sensor under UV excitation to CMM from 25 July until 23 Oct (2017). It can be observed that the response of the sensor decreases from 68% to 54% after 30 days and then is stable. After 45 days sensor has been exposed to the UV lamp with 400 W power for 2 hours and then the test was repeated again. The results showed that the response of sensing was about 90% of the initial value.

## Conclusion

In summary, we have synthesized well-aligned ZnO nanorods on p-type Si (100) substrate with wurtzite structure, average diameter 100 nm, and 600 nm length. Calculated density of ZnO NRs was ~6 × 10^9^ cm^−2^ that significantly was higher than previous reports. The prepared ZnO NRs sensor was tested to detect CMM gas. Our result showed that under UV irradiation the operating temperature decreased from 225 °C to 100 °C for ZnO NRs. The sensor at room temperature (25 °C) under UV excitation showed an acceptable response (~23%) which makes it possible to use it in coal mines. The response and recovery time of the sensor exposed to 250–1500 ppm CMM at 100 °C operating temperature were 210–57s and 142–241 s respectively. The selectivity of the sensor to CMM gas compared with CO_2_ gas was investigated. Response of the sensor to CMM gas showed no considerable change in humid (%50) condition under UV. The sensor was stable for 90 days. Hence by using the UV-irradiation compared to previous reports our results for concentration, working temperature, response time and recovery time have been improved.
